# O.5.1-7 Travel patterns and CO2 emissions of recreational league play in football and handball in Germany - an explorative study

**DOI:** 10.1093/eurpub/ckad133.237

**Published:** 2023-09-11

**Authors:** Julian Resch, Michael Dittrich, Heiko Ziemainz, Karim Abu-Omar

**Affiliations:** Friedrich-Alexander-Universität Erlangen-Nürnberg, Germany; julian.resch@fau.de; Friedrich-Alexander-Universität Erlangen-Nürnberg, Germany; julian.resch@fau.de; Friedrich-Alexander-Universität Erlangen-Nürnberg, Germany; julian.resch@fau.de; Friedrich-Alexander-Universität Erlangen-Nürnberg, Germany; julian.resch@fau.de

## Abstract

**Purpose:**

Recreational sports are commonly recommended for their health-enhancing effects. But broadening the perspective on planetary health raises the question of how they impact the environment. While there is a body of evidence on the impacts of professional sports on planetary health, there is currently a lack of studies investigating the sustainability of grassroots level sports, in particular team sports in recreational leagues. This study set out to explore travel patterns in different recreational leagues in the sports of football and handball in Germany.

**Methods:**

We derived data including club location and game schedules for amateur football and handball teams in the region of Erlangen, Germany, from official websites of sport federations. Using google maps, we calculated the travel distances for every match-up in season 2022/23 and highlighted the distribution of teams in divisions by mapping.

**Results:**

Even on a low recreational level, significant travel distances are accrued by teams over a season. In general, playing in a more competitive league results in longer travel distances for football teams. On average, women teams cover longer travel distances compared to men’s teams. Playing handball results in more travel compared to playing football. The current set-up of divisions can be improved to limit travel distances.

**Conclusions:**

Despite some limitations (e.g., number of included divisions or difficulty translating the distances into CO2 emissions), this investigation sheds light on the environmental impact of recreational league play. The sheer number of sports teams suggests the importance of investigating recreational league play and supporting sport federations to optimize game schedules to limit travel emissions.

**Funding Source:**

The study was not funded.

